# Symmetric Sensorimotor Somatotopy

**DOI:** 10.1371/journal.pone.0001505

**Published:** 2008-01-30

**Authors:** Simon A. Overduin, Philip Servos

**Affiliations:** 1 Department of Brain and Cognitive Sciences and McGovern Institute for Brain Research, Massachusetts Institute of Technology, Cambridge, Massachusetts, United States of America; 2 Department of Psychology, Wilfrid Laurier University, Waterloo, Ontario, Canada; University of Birmingham, United Kingdom

## Abstract

**Background:**

Functional imaging has recently been used to investigate detailed somatosensory organization in human cortex. Such studies frequently assume that human cortical areas are only identifiable insofar as they resemble those measured invasively in monkeys. This is true despite the electrophysiological basis of the latter recordings, which are typically extracellular recordings of action potentials from a restricted sample of cells.

**Methodology/Principal Findings:**

Using high-resolution functional magnetic resonance imaging in human subjects, we found a widely distributed cortical response in both primary somatosensory and motor cortex upon pneumatic stimulation of the hairless surface of the thumb, index and ring fingers. Though not organized in a discrete somatotopic fashion, the population activity in response to thumb and index finger stimulation indicated a disproportionate response to fingertip stimulation, and one that was modulated by stimulation direction. Furthermore, the activation was structured with a line of symmetry through the central sulcus reflecting inputs both to primary somatosensory cortex and, precentrally, to primary motor cortex.

**Conclusions/Significance:**

In considering functional activation that is not somatotopically or anatomically restricted as in monkey electrophysiology studies, our methodology reveals finger-related activation that is not organized in a simple somatotopic manner but is nevertheless as structured as it is widespread. Our findings suggest a striking functional mirroring in cortical areas conventionally ascribed either an input or an output somatotopic function.

## Introduction

Receptors in the skin provide cutaneous information to the primary somatosensory (SI) cortex. Following detailed electrophysiological studies of cortical somatosensory responses in monkeys, investigations of somatosensory organization in human cortex have either relied on superficial recordings from epileptic patients [1], or more recently from fMRI (functional magnetic resonance imaging) [2]–[5]. Previous human somatotopy studies using fMRI have been limited not only by scanning resolution [2], [4] but by an underlying premise that fMRI reveals discrete activation “hotspots” in response to cutaneous stimulation [3], [5], like those measured electrophysiologically as the firing output of cells. Here we sought cortical somatosensory responses using high-resolution (4T) fMRI and an interpretation of the measured activity as reflecting more the metabolic demands of dendritic input to cells [6].

**Figure 1:**
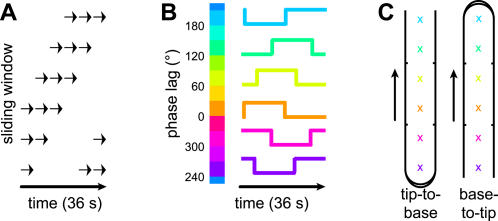
Sliding window paradigm. (A) Pneumatic stimulation was delivered to D1, D2, or D4, via a restricted set of jets within a sliding window of stimulation. (B) Voxels correlated to the reference waveform were assigned one of 12 colors, according to the phase delay giving the maximal correlation. Because the number of jet positions was only four (D1) or six (D2/D4), the color values included interpolated phase lags. (C) The colors corresponded to locations on the digit surface, although the mapping from phase delay to location differed depending on stimulation direction.

Our experimental paradigm adapted the phase analysis method used previously to identify retinotopic maps in visual cortex [7]. A reference time course consisting of periods of stimulation interspersed with non-stimulation intervals was created by a “sliding window” [8], [9] of stimulation that cycled repeatedly over the digit surface (Fig. 1). Any location on the digit surface experienced alternating windows of stochastic stimulation and silence. Neighboring locations were stimulated in like fashion, but with a stimulation time course that was staggered in time. This design was not strictly a block design, except with respect to individual locations on the digit surface. Any correlated cortical activity should have been driven by an on/off stimulation time course, but one whose particular phase lag would indicate its sensitivity to a certain location on the digit surface. The relatively slow progression of the stimulation window over the digit, and the random nature of the jet activations within any stimulation window, mitigated any “cutaneous rabbit” illusions of movement [10]. As a within-digit control, we varied the general direction of stimulation, from the base of the finger to the fingertip, or from the tip to the base.

We passively stimulated the hairless surface of the thumb (D1), index (D2) and ring finger (D4) of human subjects using puffs of air delivered through a custom-built apparatus. While stimulating the digits we measured the cortical blood-oxygenation level dependent (BOLD) response using fMRI. We assigned each functional voxel a particular location on the digit surface based on its phase lag of maximal correlation to the stimulation time course.

## Methods

### Subjects

We scanned seven healthy right-handed human subjects (four males; average age 26 years), at the Robarts Research Institute (London, ON) using a 4T magnetic resonance imager (Varian, Palo Alto, CA; Siemens, Erlangen, Germany). Incorrect slice plane positioning excluded the experiments of one subject, and a significant motion artifact excluded the D4 experiments of subject 1.

### Scanning parameters

We used a custom 14-cm quadrature surface coil centered on the left frontoparietal region to acquire functional and anatomical images, and a custom transmit-receive cylindrical birdcage coil to acquire a full-brain anatomical volume in a separate scanning session. A T2*-weighted gradient echo-echo planar imaging (EPI) pulse sequence was used in functional image acquisition (TR = 750 ms, 4 shots, TE = 15 ms, FA = 40°, FOV = 19.2×19.2 cm). Nine contiguous pseudocoronal 5-mm thick functional slice planes (1.5×1.5 mm resolution in-plane) were sampled, and 72 such volumes were collected over each 216-s experiment. A T1-weighted volume was acquired in the same session, consisting of 64 contiguous pseudocoronal 1-mm thick anatomical slice planes (0.75×0.75 mm resolution in-plane; TR = 12 ms, TI = 500 ms, TE = 6 ms, FA = 11°, FOV = 19.2×19.2 cm) approximating the orientation of each subject's central sulcus. The full-brain T1-weighted volume included 256 contiguous axial 0.94-mm thick slice planes (TR = 12 ms, TI = 500 ms, TE = 6 ms, FA = 11°, FOV = 24.0×24.0 cm).

### Stimulation equipment

We used a custom Plexiglas frame to deliver pulses of air via an array of shallow 0.5-cm wide depressions designed to conduct reflected air away from the hand surface. Connected to 16 of these depressions were flexible tubes leading to a series of manifold-mounted air valves. The pattern of these 16 connections was chosen for each subject to optimize the distribution of the active jets under the digits. In particular, the jet locations were spaced regularly about 1.5 cm apart in a straight line under D1 (4 jets), D2 (6) and D4 (6). The tubing length and aperture was consistent across channels. The air valves were computer-controlled and received air from a compressor at a steady-state pressure of 240–275 kPa. Subjects' eyes were closed; a bite bar stabilized the head.

### Experimental paradigm

In six experiments on each subject we separately stimulated three digits (D1, D2, and D4) and two directions of stimulation (tip-to-base and base-to-tip). Each subject participated in all experiments in a pseudorandom sequence such that no two successive experiments would involve the same digit. Each experiment was defined as six successive 36-s stimulation cycles. Within each stimulation cycle a “window” of stimulation (equal to half of the available jets) rotated through all the jets spanning a digit. During each 18-s half-cycle the potentiated jets were activated in a pseudorandom sequence, in bursts of between five and ten 40-ms air puffs each separated by 60 ms. Each such burst was confined to a single jet, but could be followed by another burst at the same location or at another jet potentiated within the window. These bursts were separated by random inter-burst intervals of 1–2 s. The resulting puff and burst stimulation frequencies were within the range of superficial cutaneous receptors [11].

### Anatomical analysis

Our anatomical regions of interest included areas 4, 3a, 3b, and 1. These were defined for each subject based on visual inspection of full-brain cortical anatomy. Areas 4 (along the anterior wall of the central sulcus and onto the precentral gyrus) and 3b (on the posterior wall of the central sulcus) lay facing each other across the fundus of the central sulcus (area 3a), and converged medially at the paracentral lobule. Area 1 lay on the crown of the postcentral gyrus and extended posteriorly to the rostral lip of the postcentral sulcus.

### Functional analysis

Prior to our correlation analysis, we preprocessed the functional images in BrainVoyager (Brain Innovation, 2000) within the frequency domain by removing linear trends in vascular activity and highpass filtering at 0.014 Hz (cf. [12]). Functional data were not spatially averaged. The 18-s on/off half-cycles defined a reference time course that we blurred and shifted by a 5-s hemodynamic response function. The reference time course was then shifted by iterative 3-s increments, and correlated with the functional data at each delay. Functional voxels were color-coded according to the phase lag giving maximal correlation (Fig. 1B). Note that we used twelve phase values, each corresponding to a particular 3-s shift of the 36-s stimulation cycle. This number of phase values exceeded the number of stimulation locations on the digits (four or six). We chose to use twelve phase values in order to retain the 3-s temporal resolution of the BOLD signal, and because it was the lowest common denominator of four and six and thus allowed us to use the same phase analysis parameters and color scale for all digits. After coregistering a subject's functional and anatomical slice planes, phase-coded functional data were assigned to one of the regions of interest (ROIs). Voxels above a correlation threshold (r≥0.3) were tested for uniformity of phase using Rayleigh's statistic [13].

### Cortical flattening

In order to quantify our qualitative impression that functional activity in the precentral gyrus tended to mirror that of the postcentral gyrus, we flattened the pericentral cortical area into a 2D manifold using the Isomap algorithm [14, 15; see also 16], a nonlinear dimensionality reduction algorithm. Isomap is a variant of classical multidimensional scaling (MDS [17]), and like MDS, it computes a non-sparse distance matrix for all points in the workspace to find a lower-dimensional representation of the data. Unlike MDS, the distances are not Euclidean but are the sums of the geodesic distances, which accumulate in transition from the center of one local neighborhood to the next. Isomap thus allows a highly-folded surface to be flattened even when voxels on either side of a sulcus may be nearly adjacent in Euclidean space. Isomap also does not assume that the extracted surface is an intrinsically linear 2D sheet, but instead generalizes to a larger class of nonlinear manifolds [14], which likely include the cortical surface or patches thereof [16]. For our purposes we defined the neighborhood of a voxel as its 10 nearest neighboring voxels. This neighborhood parameter ε was the minimal size that explained 99% of the variance in geodesic distance estimates, averaged across subjects [15]. Although distances along the 2D manifold are in arbitrary units and cannot be directly equated with Euclidean distances, for plotting purposes we have labeled the approximate rostrocaudal and lateromedial orientations after rotating the manifolds so that the fundus (here, a line joining the medial and lateral termini of area 3a) was vertical, primary motor cortex (MI) was shown on its left and SI on its right. By visual observation of the extracted manifolds we confirmed that the relative positions of the cortical areas were preserved.

### Symmetry analysis

Our analysis involved the following steps. 1) We plotted the functional data (above the r≥0.3 threshold) over the flattened map of the pericentral cortex, and binned the data into grid cells (arbitrarily set at 20 units^2^) tiling this surface. Within each grid cell, we took the average phase delay value of all functional voxels within the bin; if none, the grid cell was considered not “active”. 2) We computed the degree of phase similarity in each pair of active MI (area 4) and SI (3b/1) grid cells that lay an equal distance along the fundus and an equal distance either rostral or caudal to the fundus. Given our uncertainty in assigning area 3a voxels to pre- or postcentral cortex, and in the relationship of this area to MI and SI (see Discussion), we have excluded it from this calculation. The similarity values were normalized such that if two grid cells of a pair had identical phase delays, their phase similarity was 1; if the activity was in anti-phase (e.g. 0° and 180°), the similarity was −1; if phase delays were 90° and 180°, the similarity was 0.5; etc. We then computed an average symmetry score for each experiment, over all pairs of active grid cells in that experiment. 3) To determine a threshold for “significant” symmetry, we ran 1000 Monte Carlo simulations for each experiment. In each simulation, we randomly scrambled the grid cells within the pericentral map before performing step 2. We took the 95^th^ percentile of the resulting distribution of 1000 simulated symmetry scores as the threshold for declaring a true symmetry score obtained from that experiment significant.

## Results

Our fMR imaging and subsequent analysis were directed at primary sensorimotor cortex contralateral to the right hand. Using the full-brain anatomical volume from each subject we highlighted the lateromedial extent of several cortical regions, including Brodmann's areas 3a, 3b and 1 (SI), and area 4 (MI). We found in most experiments that the entire ROI was dominated by voxels having only a few of the possible phase delay values. In particular, the distributions of voxels that were correlated to the reference time course in each digit×direction stimulation condition were significantly nonuniform for both D1 and D2 experiments (p<0.01, using Rayleigh's statistic [13] for the circular distribution of r≥0.3 voxels), but only marginally for the D4 experiments (p = 0.03). Remarkably, this trend of nonuniform phase response upon D1 and D2 stimulation was true not only in each of SI areas 3b and 1 but also in MI and area 3a in between. In cortex outside of these areas, the population of correlated voxels in these experiments was not significantly tuned (p>0.05).

**Figure 2:**
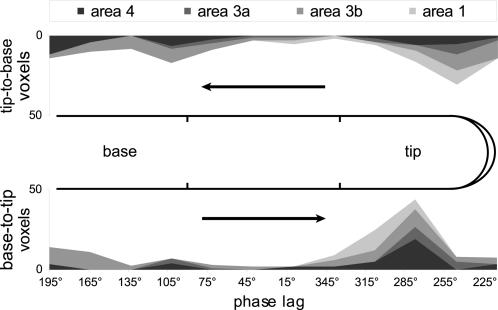
Phase value distributions were nonuniform and tuned to fingertip stimulation. Distributions of voxels (r≥0.3) are summed across digits (D1 and D2) and subjects, and are shown cumulatively for areas 4, 3a, 3b, and 1 along a linearized axis aligned with the digit surface. In the distributions for both tip-to-base (top; ordinate flipped) and base-to-tip (bottom) stimulation, there was a strong response coincident with stimulation across the fingertip jets. The peak of this response appeared to shift in phase as a function of stimulation direction.

The D1/D2 phase value distributions appeared to peak near the delay corresponding to stimulation of the digit tips (Fig. 2). Moreover, while the peaks of the tip-to-base and base-to-tip distributions were both within the phase delay range corresponding to fingertip stimulation, they were also significantly different from each other (p<0.01). Recall that each fingertip was stimulated at two locations (Fig. 1C). When the cyclical stimulation window proceeded in a tip-to-base fashion—i.e. when it contacted the most distal fingertip location before more proximal digit locations—the cortical sensorimotor activation appeared to correlate predominantly with stimulation at the more distal of the two fingertip jets (Fig. 2, top). Conversely, when the stimulation window approached the fingertip in a base-to-tip direction, the greater part of the sensorimotor activation appeared to correlate with stimulation at the base of the fingertip (Fig. 2, bottom). It thus appeared that there was an enhanced population BOLD response limited to the part of the fingertip initially contacted in each cycle of stimulation.

**Figure 3:**
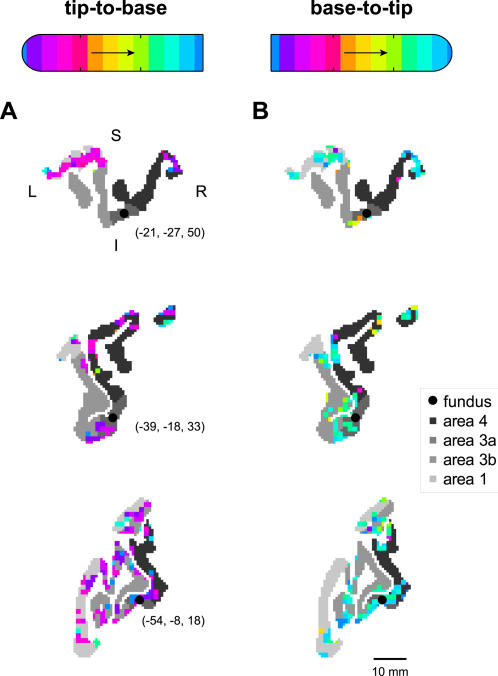
Activation tuning and symmetry in 2D slices through pericentral cortex. Three sample pseudocoronal views of sensorimotor cortex with superimposed functional activation are given for two experiments, with (A) tip-to-base stimulation of digit 2 of subject 2, and (B) base-to-tip stimulation of the same digit. In each panel, cortical areas 4. 3a. 3b. and 1 are shown in grayscale according to the legend at right. The phase delays of active voxels are shown according to the reference bars at top (see Fig. 1). Black circles and associated Talairach coordinates give the location of the fundus in these slices, estimated as the centroid of the area 3a cortex. Approximate directions L (left), R (right), S (superior) and I (inferior) are given in (A), top. A scale bar is shown in (B), bottom.

The digit-related activation in both motor and somatosensory cortices was not only extensive and biased towards fingertip representation, but was also markedly symmetric with respect to the central fundus. Such symmetry was occasionally evident in individual pseudocoronal slice planes that happened to include both pre- and postcentral gyri (Fig. 3). This symmetry was not only the result of activation clumps that spanned the banks of the central sulcus; such symmetric activation was also evident for activation on the pre- and postcentral gyral surface some distance from the sulcus (e.g. Fig. 3, top). However, the convoluted path of the central sulcus—which can cross into such 2D slices multiple times—makes it difficult to perceive the topographical distribution of the functional activity around the sulcus. Nevertheless, these images do demonstrate the tuning of the functional activity to fingertip stimulation, as can be seen in comparing the tip-to-base (Fig. 3A) and base-to-tip experiments (Fig. 3B).

**Figure 4:**
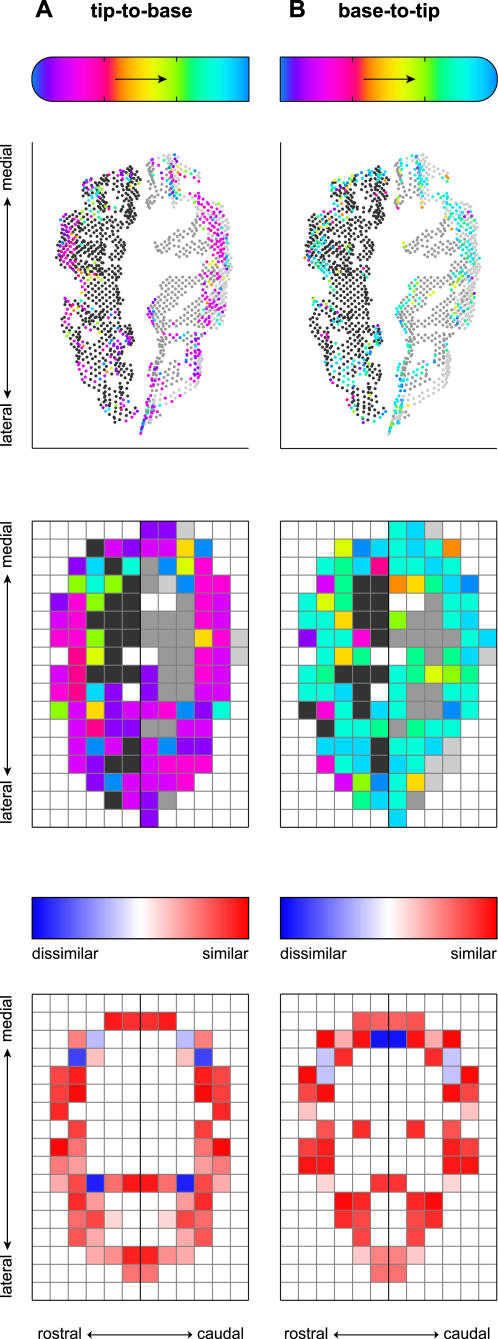
Activation tuning and symmetry in flattened representations of pericentral cortex. Shown are the same two experiments represented in Figure 3. In each plot the cortex is shown as a 2D surface, oriented in approximate rostrocaudal and lateromedial directions. The top plot shows functional data, representing phase lag according to the reference bar at top, superimposed over MI (rostral) and SI (caudal) voxels in gray, as per the legend used in Figures 2 and 3. (Area 3a, between these regions at the fundus of the central sulcus, is not shown.) The middle plot shows these same data binned into grid cells tiling the pericentral cortex. “Active” grid cells are indicated by the average phase lag across voxels within the bin. Grid cells representing pericentral cortex but lacking any active functional voxels are shown as gray (again, as per the Fig. 2 and 3 legends); cells outside MI and SI are white. The bottom plots depict the degree of similarity between active grid cells lying across from each other an equal distance from the fundus, according to the scale bar immediately above. Pairs of active grid cells with similar phase delays are both colored red; pairs of cells with activity out of phase are colored blue. These examples show widespread functional activity on both sides of the fundus, and indicate that much of this mosaic of activity was mirrored by similar activity on the opposite side of the fundus.

To visualize and quantify the symmetrical layout of the sensorimotor activation, we mapped the functional data to transformed, 2D views of the pre- and postcentral gyri (Fig. 4, top, showing the same two experiments as in Fig. 3). These flattened representations of the pericentral cortex were found using the Isomap algorithm (see Methods). We binned the functional data of MI (area 4) and SI (areas 3b and 1) into square grid cells rostral or caudal to the fundus (Fig. 4, middle). Then, we computed the degree of similarity between the averaged phase values within each pair of active grid cells lying across the fundus from each other (Fig. 4, bottom). In the experiments shown, the mean symmetry score across all such pairs of grid cells was 0.38 in (A) and 0.37 in (B). Each of these values exceeded the 95^th^ percentile of the distribution of symmetry scores obtained from 1000 Monte Carlo simulations of each experiment (see Methods), which was 0.16 in both cases.

Indeed, the average phase symmetry score across all pairs of active grid cells was in many cases significant, in comparison to the simulated data sets. In particular, in the tip-to-base and base-to-tip digit 1 experiments, we found MI/SI symmetry scores in excess of threshold in 5/6 and 5/6 subjects, respectively. In digit 2 experiments, 5/6 (tip-to-base) and 4/6 (base-to-tip) subjects demonstrated significant MI/SI symmetry. In digit 4 experiments (one of them invalidated by motion artifact; see Methods), we found significant symmetry scores in only 2/6 and 3/5 subjects. Thus while the BOLD response following stimulation at least of digits 1 and 2 may have been globally tuned to fingertip phase lags, at a finer level most of these response maps could also be characterized as relatively symmetric mosaics of activation on the two sides of the central sulcus.

## Discussion

Our experimental methodology has previously allowed us to locate discrete somatotopic maps of the digits in areas 3b and 1 [9]. These maps were defined as regions of connected voxels displaying a strong correlation to the pattern of stimulation across the digit surface, and a reversed pattern of peak correlations when the stimulation direction was reversed. However, this analysis did not reflect the larger pattern of functional activation in the region of the central sulcus. While our delineation here of areas 1, 3b, 3a, and 4 was crude for lack of precise cytoarchitectonic divisions [18], the population-level activity suggests in any case that these areas had a relatively common response to digit stimulation. Notably, the activation in each area appeared to be weighted towards fingertip stimulation of the thumb and index finger (Fig. 2), in accordance with the high sensitivity of the distal finger pad and the relative importance of D1 and D2 in precision grips and other behaviors.

Unlike areas 3b and 1, areas 3a and 4 are conventionally ascribed roles in motor output [19]. Area 3a is thought to transmit kinesthetic rather than cutaneous afferents to both motor cortex [20] and area 1 [21]. But in addition to muscle spindle input to area 3a, in monkeys cutaneous responses here can emerge with training [22] and may physically parallel those in SI [20]. The presence of somatosensory inputs to area 4 has also been neglected (as has been the motor function of parietal areas, from which a significant proportion of motor corticospinal fibers are derived, in humans [23] as in monkeys [24]). Nevertheless, single-unit recordings from awake monkeys reveal cutaneous inputs to MI that appear to segregate in modality-specific maps, with neighboring representations being differentially responsive to cutaneous and joint receptor input [25]. In humans, despite fMRI evidence that a vibrotactile stimulus can elicit precentral gyrus activity [26], [12], few researchers have tried to localize cutaneous functions to the frontal lobe.

We observed not only robust and fingertip-weighted activation on both sides of the central sulcus following stimulation of digits 1 and 2, but a symmetrical pattern to this activation. This is the first observation known to us of activation symmetry across function-defined modalities. Within-modality mirror symmetry has recently been reported within tonotopic maps of primary auditory cortex [27] and object representations within occipito-temporal cortex [28].

This symmetry was evident not only despite the different functions of SI and MI, but also despite a less orderly somatotopic organization in precentral relative to postcentral areas. For instance, in monkeys the area 4 and 3a representations appear to be more fractured than the SI maps [20], [22], perhaps consistent with the involvement of these areas in coordinated muscle recruitment. Within humans, a somatosensory homunculus has not been defined precentrally, although the motor output of MI is known to be somatotopically organized as shown by electrical stimulation in epileptic patients [1], [29].

We suggest that both non-somatotopic organization in precentral cortex and the nature of the BOLD signal may underlie previous investigators' inability to resolve discrete foci of hemodynamic activity here upon digit stimulation [9], [12]. In contrast to the spiking-defined somatotopy found with electrophysiological mapping, fMRI appears to reflect a more distributed, dendritic-level processing of neural inputs [6]. The symmetry of SI and MI responses we observed may reflect common sensory input to these areas. Although the symmetry may also reflect a diffusion of the hyperoxic response [30] in the vasculature on either side of the Rolandic artery, Young et al. [18] found that in the human left hemisphere, the resting regional blood flow within the area 3b classical “hand” area was correlated to blood flow within both anterior MI and area 3a.

In conclusion, while there may have been hotspots of BOLD response with some degree of somatotopic organization, the broader pattern of activity we observed upon cutaneous stimulation of the digits, in particular of the thumb and index fingers, was widely and symmetrically distributed on both sides of the central sulcus. In the case of thumb and index finger stimulation, the responses were also tuned to fingertip stimulation and were globally modulated by the direction of stimulation along the finger surface.

## References

[1] Penfield W, Boldrey E (1937). Somatic motor and sensory representations in the cerebral cortex of man as studied by electrical stimulation.. Brain.

[2] Blankenburg F, Ruben J, Meyer R, Schwiemann J, Villringer A (2003). Evidence for a rostral-to-caudal somatotopic organization in human primary somatosensory cortex with mirror-reversal in areas 3b and 1.. Cereb Cortex.

[3] Dechent P, Frahm J (2003). Functional somatotopy of finger representations in human primary motor cortex.. Hum Brain Mapp.

[4] Gelnar PA, Krauss BR, Szeverenyi NM, Apkarian AV (1998). Fingertip representation in the human somatosensory cortex: An fMRI study.. Neuroimage.

[5] Kurth R, Villringer K, Mackert BM, Schwiemann J, Braun J (1998). FMRI assessment of somatotopy in human Brodmann area 3b by electrical finger stimulation.. NeuroReport.

[6] Logothetis NK, Pauls J, Augath M, Trinath T, Oeltermann A (2001). Neurophysiological investigation of the basis of the fMRI signal.. Nature.

[7] Engel SA, Rumelhart DE, Wandell BA, Lee AT, Glover GH (1994). FMRI of human visual cortex.. Nature.

[8] Servos P, Zacks J, Rumelhart DE, Glover GH (1998). Somatotopy of the human arm using fMRI.. NeuroReport.

[9] Overduin S, Servos P (2004). Distributed digit somatotopy in primary somatosensory cortex.. Neuroimage.

[10] Geldard FA, Sherrick CE (1972). The cutaneous “rabbit”: a perceptual illusion.. Science.

[11] Bolanowski SJ, Gescheider GA, Verrillo RT, Checkosky CM (1988). Four channels mediate the mechanical aspects of touch.. J Acoust Soc Am.

[12] McGlone F, Kelly EF, Trulsson M, Francis ST, Westling G (2002). Functional neuroimaging studies of human somatosensory cortex.. Behav Brain Res.

[13] Fisher NI (1993). Statistical analysis of circular data.

[14] Tenenbaum JB, de Silva V, Langord JC (2000). A global geometric framework for nonlinear dimensionality reduction.. Science.

[15] Balasubramanian M, Schwartz EL, Tenenbaum JB, de Silva V, Langford JC (2002). The Isomap algorithm and topological stability.. Science.

[16] Schwartz EL, Shaw A, Wolfson E (1989). A numerical solution to the generalized mapmaker's problem: Flattening nonconvex polyhedral surfaces.. IEEE Trans Pattern Anal Machine Intell.

[17] Cox TF, Cox MAA (1994). Multidimensional scaling: Monographs on statistics and applied probability (2nd ed).

[18] Young JP, Geyer S, Grefkes C, Amunts K, Morosan P (2003). Regional cerebral blood flow correlations of somatosensory areas 3a, 3b, 1, and 2 in humans during rest: A PET and cytoarchitectural study.. Hum Brain Mapp.

[19] Mountcastle VB (1998). Perceptual neuroscience: The cerebral cortex.

[20] Huffman KJ, Krubitzer L (2001). Area 3a: Topographic organization and cortical connections in marmoset monkeys.. Cereb Cortex.

[21] Jones EG, Friedman DP (1982). Projection pattern of functional components of thalamic ventrobasal complex on monkey somatosensory cortex.. J Neurophysiol.

[22] Recanzone GH, Merzenich MM, Schriener CE (1992). Frequency discrimination training engaging a restricted skin surface results in an emergence of a cutaneous response zone in cortical area 3a.. J Neurophysiol.

[23] Jane JA, Yashon D, DeMeyer W, Bucy PC (1967). The contribution of the precentral gyrus to the pyramidal tract of man.. J Neurosurg.

[24] Russell JR, DeMeyer W (1961). The quantitative cortical origin of pyramidal axons of Macaca rhesus, with some remarks on slow rate of axolysis.. Neurology.

[25] Strick PL, Preston JB (1978). Two representations of the hand in area 4 of a primate. II. Somatosensory input organization.. J Neurophysiol.

[26] Moore CI, Stern CE, Corkin S, Fischl B, Gray AC (2000). Segregation of somatosensory activation in the human rolandic cortex using fMRI.. J Neurophysiol.

[27] Formisano E, Kim DS, Di Salle F, van de Moortele PF, Ugurbil K (2003). Mirror-symmetric tonotopic maps in human primary auditory cortex.. Neuron.

[28] Hasson U, Harel M, Levy I, Malach R (2003). Large-scale mirror symmetry organization of human occipito-temporal object areas.. Neuron.

[29] Woolsey CN, Erickson TC, Gilson WE (1979). Localization in somatic sensory and motor area of human cerebral cortex as determined by direct recording of evoked potentials and electrical stimulation.. J Neurosurg.

[30] Malonek D, Grinvald A (1996). Interactions between electrical activity and cortical microcirculation revealed by imaging spectroscopy: Implications for functional brain mapping.. Science.

